# Policy, Financing, and Regulatory Barriers to Adopting AI-Powered Electrocardiography Interpretation Clinical Decision Support System in Ethiopia: A Qualitative Study

**DOI:** 10.3390/ijerph23040520

**Published:** 2026-04-17

**Authors:** Minyahil Tadesse Boltena, Ziad El-Khatib, Amare Zewdie, Paul Springer, Abraham Tekola Gebremedhn, Tsegab Alemayehu Bukate, Yeabsira Alemu Fantaye, Mirchaye Mekoro, Mulatu Biru Shargie, Abraham Sahilemichael Kebede

**Affiliations:** 1 Artificial Intelligence Innovation Laboratory, Armauer Hansen Research Institute, Ministry of Health, Addis Ababa 1005, Ethiopia; amarezewdie23@gmail.com (A.Z.); abratekola@gmail.com (A.T.G.); tseguama@gmail.com (T.A.B.); yeabsiraalemu34@gmail.com (Y.A.F.); 2 Cochrane Ethiopia, Center of Excellence for Evidence Synthesis and Knowledge Translation, Armauer Hansen Research Institute, Ministry of Health, Addis Ababa 1005, Ethiopia; 3 Ethiopian Evidence Based Health Care Centre: A Joanna Briggs Institute Center of Excellence, Faculty of Public Health, Institute of Health, Jimma University, Jimma P.O. Box 378, Ethiopia; 4 Department of Global Public Health, Karolinska Institutet, 17177 Stockholm, Sweden; 5 MI4People gGmbH, Maxhofstraße 76, 81475 Munich, Germany; paul.springer@mi4people.org; 6 Health Poverty Action (HPA), Addis Ababa SW81SJ, Ethiopia; m.mekoro@healthpovertyaction.or.ke (M.M.); mulatubiru@gmail.com (M.B.S.); 7 Lero SFI Research Centre for Software, Health Research Institute, University of Limerick, V94NYD3 Limerick, Ireland; abraham.kebede@ul.ie

**Keywords:** artificial intelligence, electrocardiography interpretation, political economy, Ethiopia

## Abstract

**Highlights:**

**Public health relevance—How does this work relate to a public health issue?**

- AI-powered ECG technologies have the potential to address Ethiopia’s growing cardiovascular disease burden by expanding diagnostic access, provided that supportive policy, regulatory, and financing frameworks are in place.
- AI-powered ECG interpretation clinical decision support system (CDSS) has the potential to decentralize specialized cardiovascular care to primary healthcare, thereby saving lives and reducing mortality and morbidity. Its adoption goes beyond a technical issue, but a political economy issue, requiring evidence-informed decision-making, sustainable healthcare financing, and regulatory clarity. By optimizing scarce resources and ensuring higher service delivery at lower cost, this innovation can strengthen Ethiopia’s health system and make it more responsive and resilient.

**Public health significance—Why is this work of significance to public health?**

- This study provides one of the first qualitative, political economy analyses of policy, financing, and regulatory barriers to AI-powered ECG interpretation CDSS adoption in Ethiopia. It highlights how evidence-based clinical decision support can generate significant return on investment through efficient use of scarce resources, while also advancing patient-centered and human-centered care. By exposing systemic gaps in governance and financing, the study contributes to implementation science and informs strategies for seamless integration of innovation into health systems.

**Public health implications—What are the key implications or messages for practitioners, policy makers, and/or researchers in public health?**

- Findings underscore the imperative for context-specific AI policies, robust regulatory standards, and sustainable financing mechanisms to enable evidence-based healthcare. Phased implementation, continuous monitoring, and learning from prior digital health programs and projects are essential to optimize adoption, ensure efficient resource use, and strengthen health system resilience. For policymakers, healthcare practitioners, and researchers, the study demonstrates that effective adoption of AI-powered ECG interpretation CDSS requires coordinated governance, financing reforms, and integration strategies that deliver higher service at lower cost, decentralize specialized cardiovascular care, and ultimately save lives.

**Abstract:**

Cardiovascular diseases are a growing public health challenge in Ethiopia, worsened by limited access to diagnostics, including ECG, and shortages of specialized expertise. AI-powered ECG offers potential to improve diagnostic accuracy, efficiency, and access in resource-limited settings, but its adoption is influenced by policy, regulatory, financing, and governance factors, which are not well understood in Ethiopia. This study explored these system-level determinants using qualitative methods from September to October 2025 across federal institutions, four regions, and five tertiary hospitals. Twenty-five stakeholders, including policymakers, regulators, digital health experts, and hospital leaders, were interviewed. Data were transcribed verbatim, coded inductively, and analyzed thematically. Six themes emerged: policy and governance, regulatory frameworks, financing and cost considerations, data governance and bias, integration barriers, and sustainability recommendations. Findings showed AI-powered ECG interpretation aligns with Ethiopia’s digital health and noncommunicable disease priorities, but the country lacks AI-specific health policies, clear regulations, and dedicated budgets. Financing is largely donor-dependent, data governance and algorithmic bias remain concerns, and infrastructure gaps and digital skill shortages limit readiness. Study participants recommended learning from prior digital health projects, coordinated scale-up, phased implementation, and continuous monitoring. Effective adoption will require context-specific policies, sustainable financing, robust regulation, strong data governance, and careful system integration to ensure equitable, responsible, and sustainable use.

## 1. Introduction

Cardiovascular diseases (CVDs) are a leading cause of morbidity and mortality globally, with a growing burden in low- and middle-income countries (LMICs). Shortages of specialized healthcare professionals and diagnostic capacity continue to limit the timely detection and management of cardiac conditions in sub-Saharan Africa, including Ethiopia [1,2]. LMICs take a disproportionate share of the global cardiovascular disease burden, accounting for nearly 80% of the global cardiovascular deaths, which is further exacerbated by limited access to diagnostic resources, shortages of specialized healthcare professionals, and persistent infrastructure constraints [3].

Early detection and timely management of cardiac conditions rely heavily on access to electrocardiography (ECG) and expert interpretation, yet such services remain unevenly distributed and concentrated in specialized healthcare facilities [4,5]. Nowadays, an artificial intelligence-powered ECG (AI–powered ECG) is an emerging, transformative application of AI on cardiovascular disease diagnosis and treatment [6,7]. Global evidence shows that AI-powered ECG interpretation CDSS improves early detection of cardiac abnormalities and enhances the efficiency and equity of cardiovascular diagnostic services [8,9]. In this context, AI–powered ECG technologies have gained increasing attention for their potential to support clinical decision-making, enhance diagnostic accuracy, and expand access to cardiac care at lower levels of the health system [10,11].

Globally, the integration of AI into healthcare is accelerating, supported by national digital health strategies, ethical frameworks, and international initiatives that promote responsible and trustworthy AI use [12,13]. AI-powered ECG systems, in particular, are viewed as promising tools for addressing shortages of specialized experts and improving efficiency in cardiovascular care pathways [14]. However, evidence from LMICs suggests that the successful adoption of AI-based health technologies is influenced by broader health system factors, including policy environments, governance structures, regulatory readiness, and sustainable financing mechanisms [15,16].

In Ethiopia, recent national commitments to digital health transformation and noncommunicable disease (NCD) prevention provide a potentially enabling environment for the introduction of AI-enabled diagnostic tools [1,17]. Ethiopia’s Digital Health Strategy and the National NCD Prevention and Control Strategy provide a supportive policy foundation for the adoption of AI-enabled health technologies. The frameworks emphasize strengthening early diagnosis, expanding access to digital health solutions, and improving the management of cardiovascular diseases. In this context, AI-powered ECG represents a promising opportunity to accelerate progress toward these national priorities by enhancing diagnostic capacity and efficiency [17,18]. Despite these strategic priorities and growing openness and interest in health innovation, including AI-powered ECG, the absence of dedicated AI-specific policies for healthcare raises critical questions regarding policy coherence, institutional responsibility, and long-term sustainability. Regulatory clarity is particularly important for AI-powered medical technologies, given concerns related to safety, accountability, data protection, and clinical responsibility [19]. Financing represents another major determinant of AI adoption in resource-limited settings. AI-powered diagnostic tools often require upfront investment, ongoing operational costs, and sustained budgetary commitments for maintenance, training, and system upgrades [20]. In the absence of clearly defined financing mechanisms, AI initiatives risk remaining fragmented or confined to short-term pilot projects. Moreover, funding decisions are frequently shaped by perceptions of cost-effectiveness, health impact, and equity, particularly in health systems facing competing priorities and limited fiscal space [21].

Alongside policy, regulatory, and financing considerations, data governance and algorithmic bias have emerged as critical cross-cutting issues in AI deployment. The use of patient data for AI development and implementation requires robust governance frameworks to ensure privacy, appropriate data sharing, transparency, and accountability [12]. In addition, AI models trained on non-representative datasets may lead to misdiagnosis if bias is not adequately addressed, underscoring the need for contextual validation and continuous oversight [22]. Despite the growing interest in AI-enabled technologies in health care, including the AI-powered ECG in Ethiopia, there remains limited empirical evidence examining the policy, financing, and regulatory contexts that shape their integration within the health system. Understanding these multifaceted factors is essential for informing evidence-based strategies that support a responsible and sustainable integration of an AI-powered ECG tool. This formative qualitative study, therefore, aims to explore the policy, financing, and regulatory factors influencing the adoption of an AI-powered ECG interpretation CDSS tool in CVD care in Ethiopia.

## 2. Materials and Methods

### 2.1. Study Setting and Context

This qualitative study was conducted from September to October 2025. The study was conducted in selected organizations at the federal level, four administrative regions: Addis Ababa, Amhara, Central Ethiopia, and Oromia, and five territory-level hospitals: Black Lion, St. Paul’s, Felege-Hiwot, Jimma University Medical Center, and Dessie Comprehensive Specialized Hospitals. The study sites were purposefully selected to include: major stakeholders who have a great link to the integration of AI-powered ECG interpretation, facilities in need/cardiac centers/, different geographic locations, and healthcare delivery contexts.

### 2.2. Study Design

The study employed a formative qualitative study design to explore the political, financing, and regulatory factors influencing the adoption of AI-powered ECG interpretation CDSS in Ethiopia. A formative qualitative research approach was appropriate to generate in-depth, context-specific insights from key stakeholders to understand complex systems, stakeholder perspectives, and contextual factors that shape the feasibility and acceptability of the technology. This approach will enable us to explore participants’ experiences and insights to generate actionable evidence for guiding policy and programmatic strategies for AI-powered ECG integration in Ethiopia.

### 2.3. Study Participants

The study followed a purposive sample of 25 key stakeholders selected for their expertise, decision-making authority, and relevance to the integration of AI-powered ECG interpretation CDSS. The participants were drawn from three distinct categories:

Federal level stakeholders: This group comprised eight leaders from the Ethiopian Ministry of Health, specifically from departments focused on health innovation, digital health, and healthcare financing. In addition, representatives from pertinent organizations such as the Ethiopian Food and Drug Authority (EFDA), Center for Digital Health Innovations (CDHI), Ministry of Innovation and Technology, and the Ethiopian Artificial Intelligence Institute (EAII) were included. These stakeholders provided insights into policy perspectives, regulatory frameworks, funding opportunities, and strategic health initiatives.

Regional level stakeholders: Ten participants were selected from four regional states: Amhara, Oromia, Addis Ababa, and Central Ethiopia. This selection included the NCD/medical service division, health innovation experts, and digital health leaders who operate at the regional level, ensuring that the voices of different geographical contexts within Ethiopia were represented. Their perspectives were crucial in understanding how regional policies and practices could facilitate or hinder the AI-powered ECG interpretation CDSS adoption.

Tertiary hospitals: The final group consisted of seven health facility leaders, medical directors, and ICU and emergency care department heads from five tertiary-level hospitals: Black Lion Hospital, St. Paul’s Hospital, Felege-Hiwot Hospital, Jimma University Medical Center, and Dessie Comprehensive Specialized Hospital. The inclusion of these hospital representatives was aimed at capturing the experiences of health facility leaders who would put into practice the policy initiatives, be involved in regulation, and directly implement AI-powered ECG interpretation CDSS in clinical settings.

### 2.4. Sampling Method

The sampling for this study utilized a maximum variation purposive sampling technique, which is appropriate for qualitative research that seeks to capture a broad range of perspectives and experiences. In our case, participants were identified and selected based on their roles, expertise, and relevance to AI-powered ECG adoption, including policymakers, regulators, digital health and innovation experts, health financing professionals, and hospital leaders. By including stakeholders from various levels and sectors, the study aimed to illuminate the diverse influences on AI-powered ECG adoption. The decision on the final sample size was guided by the principle of data saturation, i.e., 25 participants were recruited iteratively until thematic saturation was reached when subsequent interviews no longer yielded new insights or ideas.

### 2.5. Data Collection Methods and Procedures

Data were collected through semi-structured, key informant interviews, allowing for flexibility in exploring topics relevant to the participants while ensuring that all essential questions were addressed. Interviews were conducted in a private setting to foster an open and honest dialog. The data were collected by five experienced and trained qualitative research assistants, medical doctors, and master’s degree holders who are fluent in Amharic and English. The data collectors critically examined their own biases, experiences, and how these might influence the research process and findings through self-reflection and discussion among each other. The data collectors were trained for three days on data collection techniques, interview guides, and informed consent. Each session was audio-recorded with the participant’s consent, and detailed field notes were taken to capture non-verbal cues and contextual observations that might be relevant to the analysis. Following the completion of the interviews, the recorded audio was transcribed verbatim. This meticulous transcription process ensured that participants’ narratives were accurately represented, preserving their meanings and expressions.

### 2.6. Data Management and Analysis

All voice-recorded files, including respective transcriptions and field notes, were stored in REDcap (Version 13.10.1) electronic data capture tools hosted at Armauer Hansen Research Institute (AHRI). Audio recordings were first translated from the local language into English and subsequently transcribed verbatim by experienced researchers involved in the data collection. After an initial reading of the full transcripts and a review of the interview guides, a comprehensive codebook was developed by the research team members. The codebook included the code, its definition, a representative text example from the transcripts, and the corresponding subtheme and main theme (Supplementary File S1). An inductive coding approach was applied using MAXQDA (version 2025) by AZ and MTB. The codebook served as a reference throughout the coding process and guided the presentation of the results. Thus, codes were generated inductively from the data and iteratively grouped into sub-themes based on conceptual similarities. These sub-themes were then refined through constant comparison across transcripts and collaborative discussions among the research team, leading to the development of final themes. In this way, the research team analyzed the data thematically. The findings were then reported with detailed descriptions of these themes and subthemes. We included direct participant quotes to contextualize the findings and provide readers with vivid insights.

### 2.7. Trustworthiness

To ensure the trustworthiness, i.e., the overall quality, integrity, and credibility of the findings, we aligned the data collection and analysis procedures with the four core principles of qualitative rigor: Credibility, Dependability, Confirmability, and Transferability [23]. To strengthen the credibility and dependability of the evidence, data collectors received three days of training on both the study procedures and the subjective topic. The same experienced researchers conducted the data collection, translation and transcription processes, enabling them to retain the contextual richness of the data, such as nonverbal expressions, emotions, and interactions, while capturing the participants’ narratives. Confirmability was enhanced through systematic documentation of all methodological decisions, and an audit trail was maintained for all audio recordings and their corresponding transcriptions, ensuring that the findings are firmly grounded in the data and reflect participants’ authentic perspectives rather than researchers’ assumptions.

### 2.8. Ethical Considerations

Ethical approval for the study was granted by the Armauer Hansen Research Institute (AHRI) with reference number PO-040/25/AAERC, following a thorough review by the Institutional Ethics Review Committee. Recognizing the importance of following good ethical practice in qualitative research, extensive measures were taken to ensure ethical integrity throughout the research process. Before participation, all stakeholders received a comprehensive information sheet detailing the study’s purpose, objectives, potential risks, and benefits. This transparency allowed participants to make informed decisions regarding their involvement. Written informed consent was obtained from each participant prior to the interviews, ensuring that they were aware of their rights and the voluntary nature of their participation. Confidentiality and anonymity were rigorously maintained by assigning unique identifiers to all transcripts and securely storing data, thereby safeguarding participant identities throughout the research process.

## 3. Results

### 3.1. Characteristics of Study Participants

A total of 25 decision makers who are working in different organizations that have a great influence on the integration of AI-powered ECG interpretation, including health facility leaders who are mainly involved in cardiovascular disease management, participated in this study. Most (20) were males, with an average age of 41.4 years. Regarding the organization that they represented, six of the participants were from the federal level, which included the leaders from the Ethiopian Ministry of Health and other relevant organizations, and ten were from the four regional states in Ethiopia, and the remaining seven were from five tertiary-level hospitals in the country. More than half of them (15) were second-degree holders and have a work service year of 16.7 on average (Table 1).

**Figure 1:**
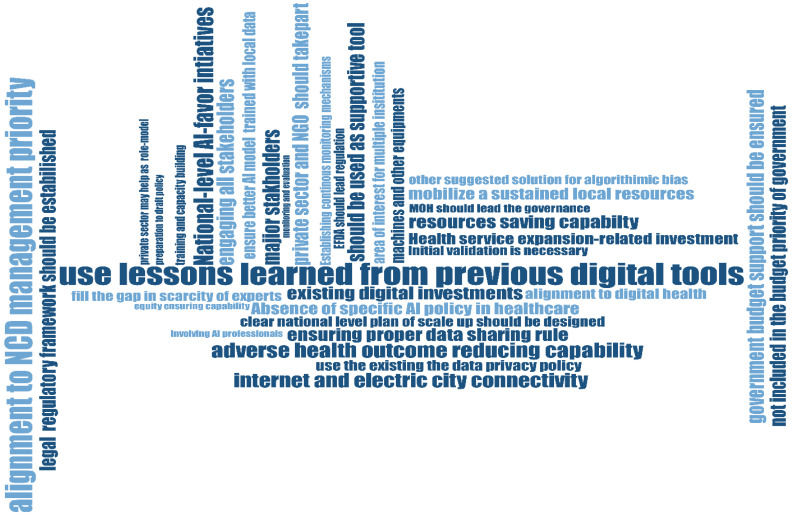
Code cloud structure generated from the data analysis in the study.

Following an exploration of the entire transcription regarding political, financing, and regulatory factors influencing national-scale AI-Powered ECG interpretation CDSS adoption in Ethiopia, over 45 codes were generated and grouped under 10 subthemes, which were further categorized into 6 main themes (Figure 1). Policy and governance contexts for AI-powered ECG interpretation, context and funding mechanism, data governance and bias mitigation concerns, anticipated integration barriers, and suggested recommendations for better functioning and sustainability were the main themes that emerged from the thematic analysis.

### 3.2. Policy and Governance Contexts for AI-Powered ECG Interpretation CDSS Integration in Ethiopia

In our study, study participants elaborated on the existing realities regarding policy and governance aspects of AI-powered ECG interpretation CDSS integration. They focused on the real-world policy, strategies, initiatives and regulatory framework that they currently practice and the need for future action. For the sake of coherence and logical elaboration, those policy and governance contexts were sub-thematized as policy perspectives and regulatory frameworks for AI-powered ECG interpretation CDSS integration.

#### 3.2.1. Existing Policy Contexts on AI-Powered ECG Interpretation CDSS Integration

Our study participants informed us about existing health technology investments in the organization they represented, particularly those associated with AI-powered ECG as a recently introduced innovation. They emphasized that those health technology efforts are mainly aimed at either health service expansion or digitalization of the health system.

*“As a health institution, we are expected to use modern technology, especially in the area of medical care. We use many types of medical equipment, including advanced machines. For example, we have imaging technologies such as MRI and CT scanners. Beyond those, we also use various laboratory machines and other medical equipment which are essential.” (A 45-year-old, male regional medical service expert)*

Participants highlighted the importance of a supportive policy environment in enabling the adoption of AI-powered ECG interpretation CDSS within the health system. They noted that while there is currently no specific AI policy dedicated to healthcare in Ethiopia, both national and international initiatives are emerging that create a favorable climate for innovation, including AI-powered ECG interpretation CDSS. Participants also mentioned that preparations are underway to draft relevant policies that could guide the safe and effective use of AI technologies.

*“I don’t think there is a specific policy in place in Ethiopia currently that talks about how to use AI. We As a responsible body must prioritize the development of an AI policy at the top to do so that it can be safely and effectively used in the healthcare system.” (A 34-year-old, female, ICU internist)*

*“Most of our health sector policy derived from the UN initiatives and declarations that makes conducive to accelerating AI adoption. This UN declaration and the initiatives through WHO endorsed by our country to develop policies, strategies, and guidelines. Therefore, it is very beneficial to facilitate this process.” (A 43-year-old, male regional medical service expert)*

Importantly, they emphasized that AI-powered ECG aligns well with existing health sector priorities, particularly in the areas of digital health transformation and NCD management. This alignment underscores the potential for AI-driven tools like AI-powered ECG interpretation CDSS to be given policy and strategy priority.

*“Nationally, as well as the biggest problem in the modern world, is non-communicable diseases. It is a neglected one. More than 53% of all deaths worldwide are due to NCDs, especially CVD and the other DM related especially retinopathy, glaucoma and other complications. This is why the problem is that, people are not treated immediately like malaria or other diseases because they have no early symptoms. When we look our diagnostic wing, including this and ECGs with AI support is beneficial. This AI-powered ECG improves the quality of care by reducing subjective viewpoints and enhancing early and objective diagnosis. As a result, it enables us to provide care to the right patient at the right time.” (A 43-year-old, male ICU internist)*

*“This AI tool definitely aligns with strategic priorities, specifically in the health sector, and using it is not optional but an obligation. In any scenario, applying this AI tool to develop prediction models, learn from big data, and support health services with technology is very helpful. Our healthcare system is growing and needs to be supported with technology to provide efficient services”. (A 36-year-old male regional digital health expert)*

#### 3.2.2. Regulatory Framework for AI-Powered ECG Interpretation CDSS Adoption

Participants emphasized that establishing a strong regulatory framework is essential for the safe and effective adoption of AI-powered ECG interpretation CDSS. They highlighted the need for clear legal and regulatory structures that define standards, safeguard patient rights, and ensure accountability. Healthcare professionals noted that the Ministry of Health should take the lead in overall governance, while the Ethiopian Food and Drug Authority (EFDA) should oversee regulation, certification, and quality assurance of the AI-powered ECG interpretation CDSS.

*“As I mentioned earlier, AI-based diagnostic tools, such as AI-powered ECG analysis, require a legal and regulatory framework for approval and use in healthcare. Generally, using AI in health and medication needs an established legal framework.” (A 33-year-old, male health innovation expert)*

*“I believe MOH should take the lion share both for adoption and implementation. MOH should prepare policy and guideline on proper utilization of and ethical use of AI in healthcare; otherwise, the implementation will be very challenging” (A 48-year-old, male digital health expert)*

*“EFDA is the one responsible for regulation under the MOH. They need to check the validity and quality of the ECG machine. We usually work in the programmatic area, so I think it would be better if they take the mandate.” (A 37-year-old female medical service expert)*

They also identified key stakeholders, including MOH, EFDA, EAII, and other relevant institutions, whose coordinated efforts will be crucial, given that AI in healthcare is an area of shared interest across multiple sectors. The study participants also elaborated the importance of a comprehensive, multi-institutional regulatory approach to guide the responsible integration of AI-powered ECG into the health system.

*“Health bureau, health facilities, service providers, innovation bureau, Artificial Intelligence institute, Information Network Security Administration (INSA) are involved. AI must be checked by Artificial Intelligence institute.” (A 41-year-old, male digital health expert)*

*“Public health institute could be part of it. I have mentioned innovation and technology. Artificial intelligence Institute, Ministry of finance for budget, Ministry of health, donation and training. For training ministry of education will come into picture. For donation partners like pharmaceutical and medical equipment suppliers.” (A 39-year-old, male ICU internist)*

### 3.3. Funding Mechanism and Related Contexts for AI-Powered ECG Interpretation CDSS Integration

Funding mechanism and its related contexts were another key area theme that our study participants deeply explored. Analysis revealed two interrelated subthemes: existing realities for funding AI-related tools and suggested mechanisms for sustainable financing, and the cost–benefit perception context influencing funding decisions.

#### 3.3.1. Existing Contexts for Funding AI-Related Tools and Suggested Mechanisms for Sustainable Financing

Participants highlighted contextual realities and challenges related to financing AI-powered tools, including AI-powered ECG interpretation CDSS. They noted that such technologies are often not yet included in government budget priorities. To address this gap, study participants emphasized the need for diversified financing approaches, suggesting active involvement of the private sector and development partners alongside public funding.

*“I think that financing for AI-powered ECG interpretation clinical decision support system, interpretation, and analysis may not yet be given high priority at the healthcare system level, since it is still a new area.” (A 45-year-old, male medical service expert)*

*“NGOs are expected to play a major role in promoting the technology once they accept it. They should help raise community awareness about its benefits and uses. Since implementing such innovations requires resources, NGOs can also provide material and financial support. Overall, their main contribution should be in promotion, advocacy, and creating public awareness about the technology.” (A 33-year-old male health innovation expert)*

Study participants also stressed the importance of mobilizing sustainable local resources, including hospital revenue collection and ensuring dedicated government budget support to reduce reliance on short-term or external funding. They underscore the need of coordinated, long-term financing mechanisms to enable the sustainable integration of AI-powered ECG interpretation CDSS into the health system.

*“AI in digital health, such as AI-powered ECG interpretation clinical decision support system, reading, and analysis, is typically financed through various sources. In our country, most health services are donor-oriented and funded primarily by NGOs from abroad. However, it is also possible to use domestic resources by engaging the community while external funding may sometimes be necessary, it is important to mobilize local resources and keep the system sustainable, rather than relying entirely on donor-oriented support.” (A 33-year-old male health innovation expert)*

*“The first one is hospital self-generated funds, as this technology will improve work efficiency and increase the number of patients who will be evaluated per day in the institution. So, patients should pay for the service provided either through community-based, social, or comprehensive insurance. Therefore, hospitals should highlight this in their strategic plan, including the budget that will be allocated for this technology from the generated income.” (A 40-year-old, male medical director)*

*“Hence digitalization is one of the funded projects specially from the government (treasury fund) as well as from other like SDG budget as digitalization is priority and the ministry is allocating budget from this unremarked budget”. (A 46-year-old, male health care financing expert)*

#### 3.3.2. Cost–BENEFIT Perception Context Influencing Funding Decisions

The study participants emphasized that perceptions of cost–benefit play a critical role in shaping health system leaders’ funding decisions for AI-powered ECG interpretation CDSS. The study participants highlighted the technology’s potential to reduce adverse health outcomes through earlier detection and timely intervention, which was viewed as a major justification for investment. They also pointed to its capacity to save resources by improving efficiency, minimizing unnecessary referrals, and optimizing the use of limited clinical expertise.

*“I believe it will enhance healthcare quality, and if so, it will influence healthcare leaders positively. As the quality of healthcare improves, other outcomes are likely to improve as well. The most important aspect is that if it enhances the services provided by healthcare facilities, reduces patient mortality, and increases the overall quality of care, it will gain support.” (A 36-year-old male digital health expert)*

*“Even though the initial investment may seem expensive, it will significantly return the capital investment over time. As mentioned earlier, the number of hours patients on queue will be greatly reduced, which is difficult to measure in monetary terms. Secondly, many lives will be saved, as early diagnosis leads to better treatment, this too is hard to quantify financially. It will also minimize the cost of medications and materials required for managing advanced disease. From the health professionals’ perspective, the time spent on a single patient will be reduced. When assisted by such devices, the number of patients evaluated can increase by 10–20 times. This demonstrates the improvement in productivity and work efficiency. From these perspectives, the return on investment is high. We need to focus on the broader benefits rather than only on the initial financial investment.” (A 37-year-old male medical director)*

Furthermore, the ability of AI-powered ECG interpretation CDSS to promote equity by extending quality diagnostic services to underserved and lower-level facilities was seen as a key benefit influencing funding considerations.

*“Additionally, accessibility is a major advantage; such technology should not be limited to cities but should be available in rural areas as well. Highlighting equity is also important, for example, access in Bahir Dar city is not the same as in more remote woreda. By focusing on equity, accessibility, resource savings, and the reduction of complications, morbidity, and mortality, leaders can be persuaded of the importance of investing in this technology.” (A 33-year-old male health innovation expert)*

### 3.4. Data Governance and Bias Mitigation for AI-Powered ECG Interpretation CDSS

The theme of data governance and bias mitigation for AI-powered ECG interpretation CDSS captures participants’ concerns about how data are managed and how algorithmic fairness is ensured in the use of AI-powered ECG diagnosis. Analysis of study participants’ perspectives revealed two closely linked subthemes: data governance issues and bias mitigation. These subthemes reflect the need for robust frameworks to guide data quality, ownership, privacy, and security, alongside deliberate strategies to identify and minimize biases that may affect model performance across different patient populations and care settings.

#### 3.4.1. Data Governance

Participants emphasized the importance of strong data governance mechanisms to ensure the safe and responsible use of an AI-powered ECG interpretation CDSS. They highlighted the need to align AI implementation with existing data privacy policies. The study participants also stressed the importance of establishing clear and transparent data protection and sharing rules to guide how patient information is accessed, used, and exchanged among stakeholders. Collectively, these measures were viewed as critical for building trust, accountability, and sustainability in the use of AI-powered ECG interpretation CDSS.

*“There are different policies, but AI is new and has only been launched at the national level. At the regional level, there are data policy laws that ensure protection, confidentiality, and accountability. When this AI tool is introduced, we will need to integrate the AI component into the existing framework.” (A 36-year-old male digital health expert)*

*“It may have an influence. If every person can access data, patient privacy may be violated. As a recommendation, the AI-powered ECG interpretation clinical decision support system should be protected by a password and should be accessible for responsible professionals only to keep patient privacy. If it has protection, it will function for a long otherwise, it may face a challenge.” (A 50-year-old female digital health expert)*

#### 3.4.2. Bias Mitigation for AI-Powered ECG Interpretation CDSS

Study participants underscored the importance of proactive bias mitigation strategies to ensure fair and reliable use of AI-powered ECG interpretation CDSS. They emphasized that developing a robust AI model trained on locally representative data is critical to improving accuracy and reducing algorithmic bias across diverse patient populations. Participants also highlighted the necessity of initial validation before widespread deployment to confirm model performance in real-world settings.

*“The first thing is to consider population variety. Many services and devices are imported from abroad. For example, in laboratory tests, reference ranges like those for CBC are based on foreign populations, not on our indigenous population. To ensure validity, it is important to include enough samples from diverse populations, rural versus urban, people with and without comorbidities, different genders, ages, height, geography, altitude, and other relevant factors, and train the system with these samples.” (A 42-year-old male ICU head)*

*“There is a heterogeneous community. To serve a heterogeneous community, the AI-powered ECG interpretation clinical decision support system should be practiced or checked on a small sample size. Then, filling gaps and strengthening based on results. Like a quality improvement program, it should be started on a small scale and scaled up to a larger place based on effectiveness is better.” (A 34-year-old female ICU head)*

Additionally, they stressed that AI-powered ECG interpretation CDSS should be used as a supportive clinical tool rather than relied upon exclusively, allowing human judgment to address potential limitations of the algorithm. Participants also pointed to the need for other complementary solutions to detect and correct bias over time, reinforcing the importance of continuous evaluation and refinement to promote equitable and trustworthy AI-assisted care.

*“AI should support health professionals. The role of AI technology is not to replace physicians but to enhance their efficiency. AI results may need to be cross-checked and should not be considered the final decision. It can be particularly useful for screening different ECGs, while physicians provide live review and refinement as needed.” (A 37-year-old male medical director)*

*“The main problem of AI in ethics is misrepresentation. So, when we feed the data to the algorithm, it shouldn’t be biased to one side of the group. We need to feed the data to the algorithm in a balanced manner to avoid algorithmic bias, it could be gendering wise, ethnicity-wise and other sociodemographic characteristics. If the question is who should oversee this algorithmic bias, there has to be a team of data engineers who could oversee such things.” (A 42-year-old male ICU head)*

### 3.5. Expected Integration Barriers of AI-Powered ECG Interpretation CDSS

Participants identified several anticipated barriers that could hinder the effective integration of AI-powered ECG interpretation CDSS into routine clinical practice. The study participants emphasized that unreliable internet and electricity connectivity remain major challenges, particularly in resource-limited settings, as these are essential for the consistent functioning of AI-based systems. They also highlighted limitations related to the availability and maintenance of ECG machines and other required equipment, like computers, which are needed for real-world application of the technology.

*“As I have told you earlier, AI tools demand various infrastructures such as internet connectivity, as well as electric power, and these things must be handled in parallel if we think that we will reach remote areas on this project, and internet connectivity and access to electricity are being improved as a nation” (A 43-year-old male medical director)*

*“I have participated in national assessment and we have limited infrastructure, and we have a shortage of machines, computers, tablets and storage devices especially in radiology service. I believe this will be challenges to implement this AI powered ECG interpretation clinical decision support system.” (A 46-year-old male digital health expert)*

In addition, gaps in training and capacity building were noted as significant barriers, as inadequate skills and technical support could reduce confidence and proper utilization of the technology.

*“We need to assess digital literacy assessment, and we must work on the implementation. Institutions like higher education and MOH must provide support in capacity building for the successful implementation of this project.” (A 46-year-old male digital health expert)*

### 3.6. Recommendation for Better Functioning and Sustainability of AI-Powered ECG Interpretation CDSS

Participants proposed several strategic recommendations to enhance the effective functioning and long-term sustainability of AI-powered ECG interpretation CDSS. They emphasized the importance of drawing on lessons learned from previous digital health tools to avoid repeated challenges and inform better implementation strategies. The study participants highlighted the need for a clear, nationally coordinated scale-up plan that engages all relevant stakeholders to ensure ownership and alignment across the health system.

*“The first lesson learned from the rollout of digital health tools is the importance of conducting a robust pilot test with the involvement of multiple stakeholders. The testing phase should be thorough, including user acceptance analysis and addressing the feedback provided. If all of this is properly executed, the implementation is likely to be successful. In addition, stakeholder involvement should include both internal and external participants. Engaging stakeholders outside the health sector, such as ENSA and the Science and Technology Institute, during pilot testing can provide diverse perspectives and help ensure the successful implementation of the tool.” (A 36-year-old male digital health expert)*

*“There may be lessons. When a new technology is introduced, you move forward by learning from past experiences. You develop the important aspects and correct the faults that existed before. As you mentioned, there is telemedicine, which is still functional now. By advancing such technologies, we can move closer to more advanced systems like AI-powered tools.” (A 45-year-old male medical service expert)*

*“As to my opinion before implementing AI, at ministry level we must have governance structure which have clear roles and responsibility and we must prepare policies and strategies by involving different stakeholders. After that we can easily implement and sustain. Technical advisory must be established and we need to develop architecture on how to integrate AI in healthcare system.” (A 36-year-old male health innovation expert)*

Establishing continuous monitoring mechanisms was also viewed as essential for tracking performance, identifying gaps, and guiding ongoing improvements. In addition, participants stressed the value of widespread awareness creation and effective information dissemination to promote understanding, acceptance, and consistent use of AI-powered ECG interpretation CDSS across different levels of healthcare.

*“Additionally, continuous monitoring is essential. For instance, during implementing AI-powered ECG interpretation clinical decision support system, indirect monitoring and evaluation should be conducted. Once approved and deployed, regular inspections are necessary since it is a machine-based system that requires frequent checks to ensure proper and safe operation.” (A 37-year-old male medical director)*

*“The first thing, it’s better to work on ideology. It is good to create awareness on the importance of AI. Second, it needs trained professionals in AI. There may be a training gap, so it is better to solve this problem. The big thing is to make understanding wide. These gaps may become the challenges we face.” (A 45-year-old male medical service expert)*

Across stakeholder groups, participants’ perspectives consistently reflected and were shaped by key system-level determinants influencing the integration of AI-powered ECG interpretation CDSS. Hospital-level leaders/Clinicians/primarily emphasized practical concerns related to infrastructure readiness (e.g., availability of reliable electricity, devices, and digital connectivity), workflow integration, and workforce capacity, highlighting how these factors affect day-to-day usability and linking successful adoption to institutional capacity. Policymakers and regulators at federal and regional levels emphasize the importance of specific policy, governance frameworks, standards, and accountability mechanisms, particularly in relation to data protection and quality assurance. Across all groups, sustainable funding for the technology was raised as a cross-cutting determinant, together with health system capacity, training, and regulatory clarity.

## 4. Discussion

This study explored the multifaceted policy, financing, regulatory, and governance factors influencing the adoption of AI-powered ECG interpretation CDSS in Ethiopia from the perspectives of multiple stakeholders. The findings highlight that while AI-powered ECG interpretation CDSS is perceived as a promising innovation for strengthening cardiovascular care, its adoption is shaped by complex systemic conditions that extend beyond technical performance alone.

### 4.1. Policy Alignment and Strategic Readiness

This study highlights the perceived importance of a supportive policy environment in facilitating the adoption of AI-powered ECG interpretation CDSS within Ethiopia’s health system. Although Ethiopia currently lacks a dedicated artificial intelligence policy for healthcare, the presence of emerging national and international initiatives, including Digital Ethiopia 2030, creates a conducive environment for health innovation, such as an AI-powered ECG tool [24]. This finding aligns with global evidence indicating that policy readiness is a critical enabler for the safe, ethical, and effective integration of AI technologies into health systems, particularly in LMICs [25]. Participants’ recognition of ongoing preparations to draft AI-related policies reflects Ethiopia’s broader engagement with global digital health and AI governance frameworks. This implies that strengthening and aligning emerging national digital and AI policy frameworks is essential to enable the safe, effective, and scalable integration of AI-powered ECG technologies within Ethiopia’s health system.

Internationally, the WHO has urged member states to develop national strategies and regulatory mechanisms to guide the use of AI in health, emphasizing transparency, accountability, and patient safety [12]. Importantly, the findings revealed that AI-powered ECG aligns with existing health sector priorities, particularly digital health transformation and the growing burden of NCDs. CVDs, which account for a substantial proportion of NCD-related morbidity and mortality globally and in Ethiopia, require timely and accurate diagnosis for effective management [26]. The perceived ability of AI-powered ECG interpretation CDSS to enhance early detection, reduce diagnostic subjectivity, and support clinical decision-making greatly aligns with the national NCD management goal and has special implications for resource-constrained settings [1,27]. Participants also framed AI-powered ECG interpretation CDSS as a tool that supports equity and efficiency by enabling “the right care for the right patient at the right time.” This perspective aligns with the Ethiopian digital health initiatives in a context where access to cardiology expertise is limited; AI-powered ECG interpretation CDSS could help bridge diagnostic gaps and support frontline clinicians [17,28]. Overall, this finding suggests that evolving global initiatives to AI in health care, combined with strong alignment between AI-powered ECG interpretation CDSS and the Ethiopian health priorities, further amplify the need to give policy priority to have a clear, context-specific, and guiding AI policies for sustainable and ethical use of AI in the Ethiopian health system. This does not necessarily imply that policy development should begin from scratch; rather, it would be more effective to leverage and build upon existing policies related to health system digitalization tools and non-communicable disease management strategies in the country. Given the strong alignment between these frameworks, such an approach would help avoid duplication of efforts and support more coordinated, efficient, and feasible integration.

### 4.2. Regulatory Frameworks and Institutional Roles

This study underscores the central role of a robust regulatory framework in enabling the safe and effective adoption of AI-powered ECG within Ethiopia’s health system. AI-based diagnostic tools require clear legal and regulatory structures to define standards, protect patient rights, and ensure accountability. This finding aligns with the global consensus that regulation is a foundational prerequisite for trustworthy AI deployment in healthcare, particularly for clinical decision-support technologies that directly influence diagnosis and treatment [12,28]. Healthcare professionals highlighted that the Ethiopian Ministry of Health should lead the overall governance, including policy development, strategic direction, and ethical guidance. Participants also identified the Ethiopian Food and Drug Authority (EFDA) as the key body responsible for regulation, certification, and quality assurance of the AI-powered ECG interpretation CDSS. EFDA’s role encompasses technical approval (pre-market evaluation), quality supervision (compliance and standards enforcement), and follow-up monitoring (post-market surveillance), while the MoH maintains a stewardship and coordination role across the system. This finding mirrors international regulatory practice, where AI-based medical devices are led by established health system governance models in which the ministries play a stewardship role and are overseen by national regulatory authorities to ensure safety, effectiveness, and quality [29]. Beyond MOH and EFDA, participants stressed the need for a multi-institutional regulatory approach involving stakeholders such as the Ethiopian Artificial Intelligence Institute (EAII), Information Network Security Administration (INSA), public health institutions, innovation and technology bodies, and education and finance sectors. This reflects the inherently cross-cutting nature of AI governance [30]. Overall, these findings indicate that AI-powered ECG interpretation CDSS needs a multi-stakeholder governance model with a shared responsibility that is led by the MOH, operationalized through EFDA, and supported by different stakeholders, like data security, innovation, and education institutions, to address the complex ethical, legal, and technical challenges and for the responsible integration of AI-powered ECG interpretation CDSS into Ethiopia’s health system.

### 4.3. Financing Realities and Cost–Benefit Considerations

This study identifies financing as a critical determinant of the successful integration of AI-powered ECG interpretation CDSS into the health system. AI-powered ECG interpretation CDSS is not yet firmly embedded within routine government budget priorities and remains largely donor dependent. While non-governmental organizations (NGOs) and development partners were seen as important catalysts for early adoption, there was strong consensus that reliance on external funding alone is insufficient for long-term sustainability. This aligns with global digital health experience, which shows that donor-driven innovations often face scale-up and sustainability challenges once pilot funding ends [31]. Importantly, participants emphasized the need to mobilize domestic resources, including government budget allocations, hospital self-generated revenue, and health insurance mechanisms. This reflects a growing recognition that AI-powered diagnostics should be integrated into routine service delivery and financing structures rather than treated as stand-alone innovations. Evidence from health financing literature suggests that embedding digital technologies into existing payment and budgeting systems is essential for institutionalization and sustainability [32]. Moreover, experiences from previous AI-based digital tools and health innovations illustrate a range of viable financing approaches, including public–private partnerships, donor-supported pilot programs, and integrating into existing health financing schemes. These approaches are particularly effective when implemented in a phased manner, initially supported by international partners and gradually transitioning to domestic financing to ensure long-term sustainability [33,34]. For Ethiopia, this implies that AI-powered ECG technologies could strategically build on existing financing structures and partnerships, leveraging early external support while progressively strengthening local investment and ownership. Such an approach would enhance feasibility, reduce financial risk, and support sustainable scale-up within the national health system.

### 4.4. Bias Mitigation and Data Governance

This study highlights bias mitigation as a central ethical and technical consideration for the adoption of AI-powered ECG in the health system. A key finding was: the use of locally representative and diverse training data to improve model validity, the need for initial validation and phased implementation before large-scale deployment, and the suggestion that AI-powered ECG interpretation CDSS should function as a decision-support tool rather than a replacement for clinical judgment as the main bias mitigation strategies. This perspective aligns with a growing body of evidence indicating that AI systems trained on non-representative datasets may perform poorly across diverse populations, the importance of local validation of AI tools, and maintaining clinician oversight [10,12,19,22]. The finding implies that in the Ethiopian context, characterized by substantial rural–urban variation, ethnic diversity, and geographic heterogeneity, locally trained, initially validated AI-powered ECG interpretation CDSS models that are overseen by clinicians may be particularly important for ensuring accurate and equitable clinical performance.

### 4.5. Integration Barriers and Pathways to Sustainable Implementation of AI-Powered ECG Interpretation CDSS

This study identified several anticipated barriers that may hinder the effective integration of AI-powered ECG interpretation CDSS into routine clinical practice, alongside stakeholder-driven recommendations to support sustainable implementation. Participants consistently identified unreliable electricity and internet connectivity, shortages and maintenance challenges related to ECG machines, computers, tablets, and data storage infrastructure, and gaps in digital literacy and technical skills as major barriers, particularly in resource-limited and remote settings. Those challenges reflect the prior digital health tools observed in many LMICs, where infrastructure limitations have constrained the scale-up of digital health interventions [31,35]. Furthermore, participants recommended learning from previous digital health tools to avoid repeating known challenges and ensuring a clear, nationally coordinated scale-up plan, continuous monitoring and evaluation, and widespread information dissemination and awareness creation for sustainable integration of AI-powered ECG interpretation CDSS in Ethiopia. This mirrors implementation science principles, which stress the importance of adaptive learning, a clear plan, and continuous improvement in scaling digital innovations [36]. As practical recommendations, it is important to align AI data governance with existing EMR data security practices in Ethiopia. Current digital health systems already apply fundamental safeguards such as role-based access control, data encryption, and restricted data sharing protocols, which can serve as a foundation for AI-powered tools [37,38]. Furthermore, leveraging existing experiences in digital health and health innovations will help integration in a secure, efficient, and scalable manner. Overall, these findings suggest that the successful and sustainable integration of AI-powered ECG interpretation CDSS in Ethiopia will depend on addressing foundational infrastructure and workforce constraints while adopting a phased, well-governed, and learning-oriented implementation approach.

## 5. Limitations of the Study

While this qualitative study provides valuable insights into the adoption of AI-powered ECG technology, several limitations must be acknowledged. First, the purposive sampling focused heavily on high-level officials and tertiary hospital leaders, potentially excluding the practical, day-to-day challenges experienced by frontline clinicians and technical staff. For example, perceptions of financing mechanisms and implementation priorities may differ between decision-makers and frontline staff. Second, the geographical focus on specific regions and major cities limits the generalizability of the findings to primary healthcare settings in remote or rural areas of Ethiopia. Third, the significant gender imbalance among participants, with a majority being male, suggests that diverse perspectives regarding the technology’s adoption may be underrepresented, such as workload implications and access to resources. Furthermore, as is common in stakeholder interviews, there is a potential for social desirability bias, where participants might align their responses with official policy goals rather than operational realities. Finally, although meticulous translation was performed, the process of translating complex qualitative data from Amharic to English inevitably risks the loss of subtle cultural and contextual nuances.

## 6. Conclusions

This study demonstrates that the adoption of AI-powered ECG in Ethiopia is shaped by interconnected policy, regulatory, financing, and governance factors. As a new and emerging technology, AI-powered ECG interpretation CDSS in Ethiopia lacks specific policy and regulatory frameworks and has not yet been included in national health budget priorities.” Integrating AI-powered ECG aligns with Ethiopia’s digital health and NCD priorities, underscoring the need for dedicated AI policies, coordinated regulatory oversight, and sustainable financing beyond pilot programs. Ensuring accurate and equitable AI-powered ECG performance requires robust data governance, local validation to mitigate algorithmic bias, representative AI models, and positioning the technology as a supportive clinical tool, alongside addressing infrastructure and workforce constraints for sustainable long-term integration. Overall, this study contributes context-specific evidence to the growing body of literature on AI adoption in LMIC health systems and, practically, the findings guide policymakers, regulators, and implementers in addressing the requirements at their respective levels. Future research should examine clinical efficacy, real-world impact, and explore strategies for scalable and sustainable AI adoption.

## Figures and Tables

**Table 1:** Characteristics of study participants included in the study.

Variable	Category	Value
Age in years	Average	41.4
Sex	Male	20
	Female	5
Organization	Ministry of Health: health innovation, digital health, health care financing	5
	Partner organizations (EFDA, CDHI, EAII)	3
	Regions: Amhara, Oromia, Addis Ababa, and Central Ethiopia Regions (4 regions’ medical service, health innovation, digital health leads)	10
	Hospital medical directors	3
	Hospital ICU and emergency department lead	4
Educational level	First degree	2
	Second degree	15
	Third degree and above	8
Work experience in years	Average	16.7

## Data Availability

All relevant data are within the manuscript and its Supporting Information Files.

## Supplementary Materials

The following supporting information can be downloaded at: https://docs.google.com/document/d/109OXCbdjeXMgb2i7UY8H4-XZdhHdQ_gM/edit?usp=drive_link&ouid=107046706163683278123&rtpof=true&sd=true (accessed on 25 December 2025). Supplementary File S1: Codebook developed for the study.

## Abbreviations

The following abbreviations are used in this manuscript

AI	Artificial intelligence
CBC	Complete blood count
CDHI	Center for Digital Health Innovations
CDSS	Clinical Decision Support System
CVD	Cardiovascular disease
EAII	Ethiopian artificial intelligence institute
ECG	Electrocardiography
EFDA	Ethiopian food and drug authority
EMR	Electronic medical record
LMICs	Lower and middle-income countries
NCD	Noncommunicable disease
SDG	Sustainable development goal
WHO	World Health Organization
